# *Goniothalamusroseipetalus* and *G.sukhirinensis* (Annonaceae): Two new species from Peninsular Thailand

**DOI:** 10.3897/phytokeys.184.73210

**Published:** 2021-10-25

**Authors:** Charan Leeratiwong, Piya Chalermglin, Richard M. K. Saunders

**Affiliations:** 1 Division of Biological Science, Faculty of Science, Prince of Songkla University, Hat Yai, Songkhla, 90112, Thailand Prince of Songkla University Hat Yai Thailand; 2 Agricultural Technology Department, Thailand Institute of Scientific & Technological Research, 35 Technopolis, Liap Khlong Ha Road, Khlong Luang District, Pathum Thani Province 12120, Thailand Thailand Institute of Scientific & Technological Research Pathum Thani Thailand; 3 Division of Ecology & Biodiversity, School of Biological Sciences, The University of Hong Kong, Pokfulam Road, Hong Kong, China The University of Hong Kong Hong Kong China

**Keywords:** Annonaceae, *Goniothalamusroseipetalus*, *Goniothalamussukhirinensis*, new species, Thailand

## Abstract

Two new *Goniothalamus* species (Annonaceae), *G.roseipetalus***sp. nov.** and *G.sukhirinensis***sp. nov.**, are described from the southern limits of Peninsular Thailand (Narathiwat and Yala Provinces). Both new species resemble *G.macrophyllus*, *G.scortechinii* and *G.uvarioides*. The addition of these two new species brings the total number of *Goniothalamus* species in Thailand to 27. Separate identification keys are provided for flowering and fruiting specimens of the Thai species.

## Introduction

The genus *Goniothalamus* (Blume) Hook.f. & Thomson (Annonaceae subfam. Annonoideae tribe Annoneae: Chatrou et al. 2012; Guo et al. 2017) is widely distributed in lowland and submontane tropical forests across Southeast Asia (Thomas et al. 2017). It is characterised by pendent, protogynous flowers with two trimerous petal whorls, with the inner whorl forming a mitriform dome over the reproductive organs (a ‘type III’ chamber *sensu*Saunders 2010). The outer petals are typically larger than the inner and periodically block the apertures between the inner petals, thereby controlling pollinator access and enabling the flower to temporarily trap the pollinating beetles (Lau et al. 2016). The timing of the petal movements that regulate pollinator trapping and release are synchronised with the circadian rhythms of the beetles (Lau et al. 2017; Saunders 2020); this allows the plant to utilise beetles with diverse circadian activities, and also allows the staminate floral phase to be extended to promote pollen deposition and enhance interfloral movement of beetles. These floral characteristics provide a possible biotic explanation for the statistically significant increase in the evolutionary diversification rate recently reported for the genus (Xue et al. 2020).

*Goniothalamus* fruits are apocarpous, with distinct fleshy ‘monocarps’ that develop from individual carpels after fertilisation. Two contrasting seed dispersal systems have been inferred, correlated with differences in fruit and seed morphology (Tang et al. 2015a): the species that are dispersed by non-volant mammals typically have ramiflorous or cauliflorous fruits with large (often sessile) monocarps and hairy seeds; whereas the species that are bird-dispersed have fruits that are borne on young growth and have small stipitate monocarps with glabrous seeds.

*Goniothalamus* is comparatively species-rich, with over 130 species. Although the genus has never been comprehensively revised, there are several recent regional taxonomic studies, including Thailand (Saunders and Chalermglin 2008), Peninsular Malaysia (Saunders 2003), Sumatra (Saunders 2002) and Borneo (Turner 2014). Twenty-five *Goniothalamus* species have been recorded from Thailand (Saunders and Chalermglin 2008), with the majority (14 species) occurring in Peninsular Thailand, viz. *G.expansus* Craib, *G.giganteus* (Wall. ex) Hook.f. & Thomson, *G.latestigma* C.E.C.Fisch., *G.macrophyllus* (Blume) Hook.f. & Thomson, *G.malayanus* Hook.f. & Thomson, *G.ridleyi* King, *G.rotundisepalus* M.R.Hend., *G.scortechinii* King, *G.tapis* Miq., *G.tavoyensis* Chatterjee, *G.tenuifolius* King, *G.tortilipetalus* M.R.Hend., *G.undulatus* Ridl. and *G.uvarioides* King. Several other *Goniothalamus* species are recorded from Peninsular Malaysia, close to the Thai border (Saunders 2003), viz. *G.curtisii* King, *G.montanus* J.Sinclair and *G.subevenius* King. Recent fieldwork in Narathiwat and Yala Provinces of Peninsular Thailand has resulted in collections of two new species that are described here as *G.roseipetalus* and *G.sukhirinensis*. The species descriptions provided here are based on observations and measurements from living material.

## New species descriptions

### Goniothalamus roseipetalus

Taxon classificationPlantaeMagnolialesAnnonaceae

Leerat., Chalermglin & R.M.K.Saunders sp. nov.

A614336C-5EED-5E2D-8515-F4E67C0210EE

urn:lsid:ipni.org:names:77221293-1

Figs 1 , 2 , 3

#### Diagnosis.

*Goniothalamusroseipetalus* resembles *G.scortechinii* and *G.uvarioides* but is distinguished by its leaves with generally fewer secondary veins (15–22 pairs), wider sepals (24–35 mm), and wider inner petals (8–11 mm). It is also distinguished from *G.scortechinii* by its wider outer petals (14–25 mm), and is distinguished from *G.uvarioides* by its smaller, single-seeded monocarps (8–15 by 7–9 mm), borne on shorter stipes (3–5 mm).

**Figure 1.**
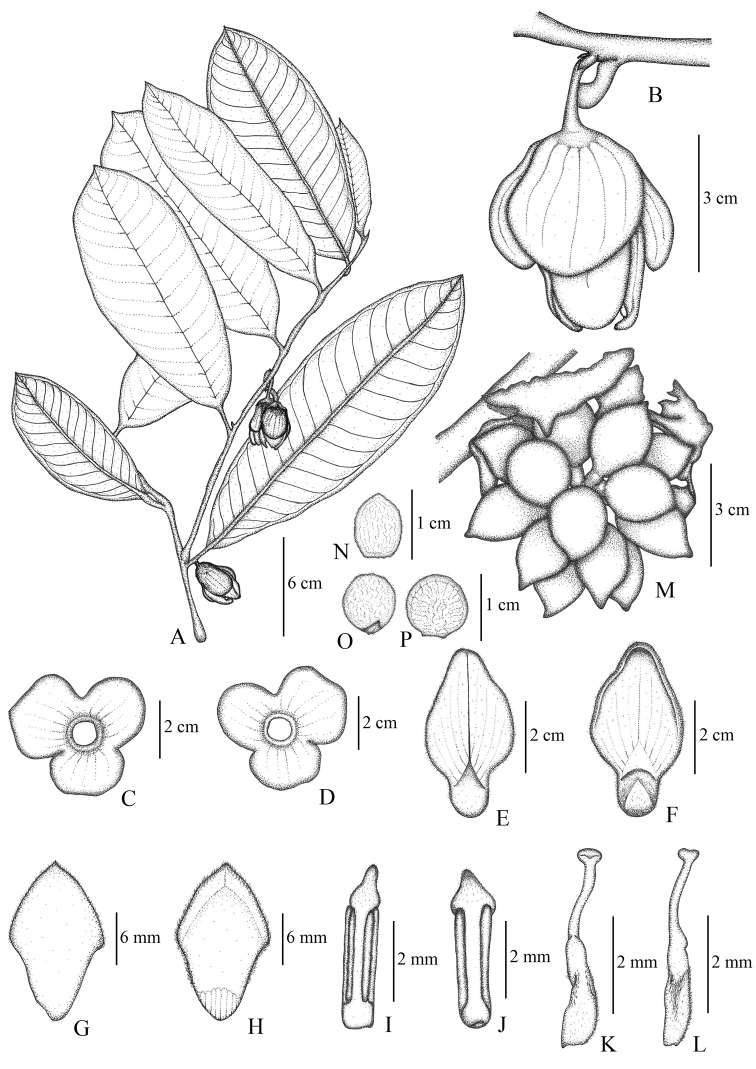
*Goniothalamusroseipetalus* sp. nov. **A** flowering branch **B** flower **C** calyx of fused sepals (abaxial) **D** calyx of fused sepals (adaxial) **E** outer petal (abaxial) **F** outer petals (adaxial) **G** inner petal (abaxial). **H** inner petal (adaxial) **I** stamen (abaxial) **J** stamen (adaxial) **K** carpel (abaxial) **L** carpel (adaxial) **M** fruit, composed of separate monocarps. **N–P** seeds (different orientations). Drawn by A. Somphrom **A–L** from *C. Leeratiwong 21*–*1708* (PSU) **M–P** from *C. Leeratiwong 21*–*1707* (PSU).

#### Types.

**Thailand**: Narathiwat: Cha Nae, Du Son Yo subdistrict, 400 m alt., 15 April 2021, *C. Leeratiwong 21*–*1706* (holotype PSU; isotypes BKF, KKU).

**Figure 2.**
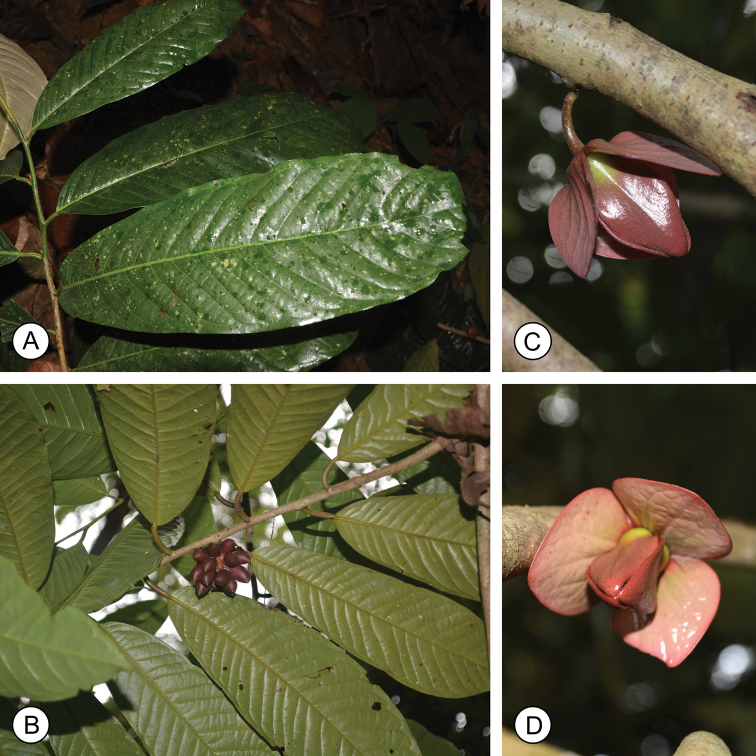
*Goniothalamusroseipetalus* sp. nov. **A** branch with leaves (adaxial) **B** fruiting branch with leaves (abaxial) **C, D** flowers. Photos by P. Chalermglin.

#### Description.

Shrubs to small trees, to 4 m. Young branches glabrous. Leaf laminas 15–40 by 3–13 cm, length/width ratio 2.8–5, elliptic to oblanceolate, apex generally acuminate (rarely acute to obtuse), acumen 3–10 mm long, base broadly cuneate, chartaceous, glabrous ab- and adaxially (sometimes sparsely pubescent over midrib); midrib strongly prominent abaxially, sunken adaxially; secondary veins 15–22 pairs, plane adaxially; tertiary veins percurrent, slightly distinct, lacking a ‘granular’ appearance abaxially; petioles 12–22 mm by 1.5–2.5 mm, glabrous to sparsely pubescent. Flowers solitary, often on main trunk (cauliflorous), rarely on older branches (ramiflorous), pendent; flowering pedicels 10–17 mm long, sparsely hairy; pedicel bracts ovate to broadly lanceolate, 2–4 by 2–3 mm. Sepals (violet-)pink, broadly ovate, 20–30 by 24–35 mm, basally connate (10–17 mm from base), apex rounded, glabrous ab- and adaxially, with sparsely hairy margins, venation distinct, 5–7-veined. Outer petals greenish-pink when young, (violet-)pink (green at claw) when mature, 25–45 by 14–25 mm with 4–10 mm-long claw, length/width ratio 1.7–2.2, fleshy, (lanceolate-)ovate, apex obtuse to mucronate, reflexed, sparsely hairy abaxially (more densely along margins basally), sparsely hairy (more densely apically) adaxially with velutinous basal region facing apertures between inner petals, midrib and venation indistinct ab- and adaxially. Inner petals 12–20 by 8–11 mm with 2–5 mm-long claw, length/width ratio 1.5–1.8, oblanceolate, densely hairy ab- and adaxially, greenish-pink when young, pale pink when mature, apex acute, lacking a glabrous lasteral flange on the inner petal claws. Stamens numerous, narrowly oblong, 3–4 mm long; connectives apiculate, papillate. Carpels 20–35 per flower, ovary oblong, 2–2.5 mm long, with white hairs; stigma and pseudostyle 2–3 mm long, stigma subulate, glabrous. Fruits with persistent calyx, immature fruits greenish-pink, mature fruits (pinkish-)red; fruiting pedicels 10–20 by 2–2.5 mm, sparsely hairy to glabrous. Monocarps 5–20 per fruit, 1–2-seeded, 8–17 by 7–10 mm, length/width ratio 1.1–1.7, ellipsoid to ovoid, apex apiculate, apicule 0.5–1.5 mm long, smooth, sparsely hairy, glossy, pericarp 1–2 mm thick, stipes 3–6 by 1.5–2 mm, moderately hairy. Seeds with mucilage, 9–11 by 8–9 mm, length/width ratio 1.1–1.6, ovoid, testa sparsely pubescent, rugose.

**Figure 3.**
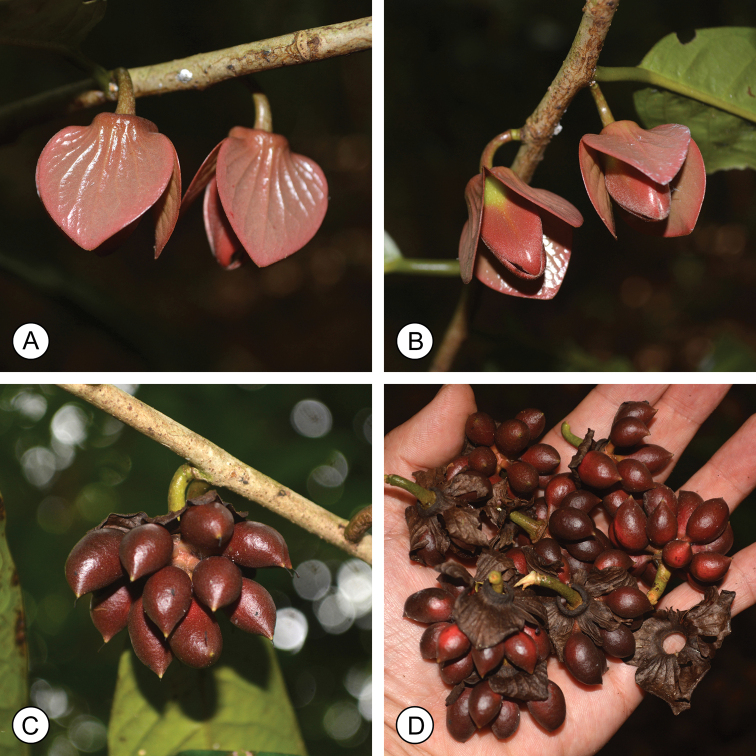
*Goniothalamusroseipetalus* sp. nov. **A, B** flowers **C, D** fruits showing persistent calyx. Photos by P. Chalermglin.

#### Phenology.

Flowering in March and April; fruiting in August (based on limited data).

#### Distribution and habitat.

**Figure 4.**
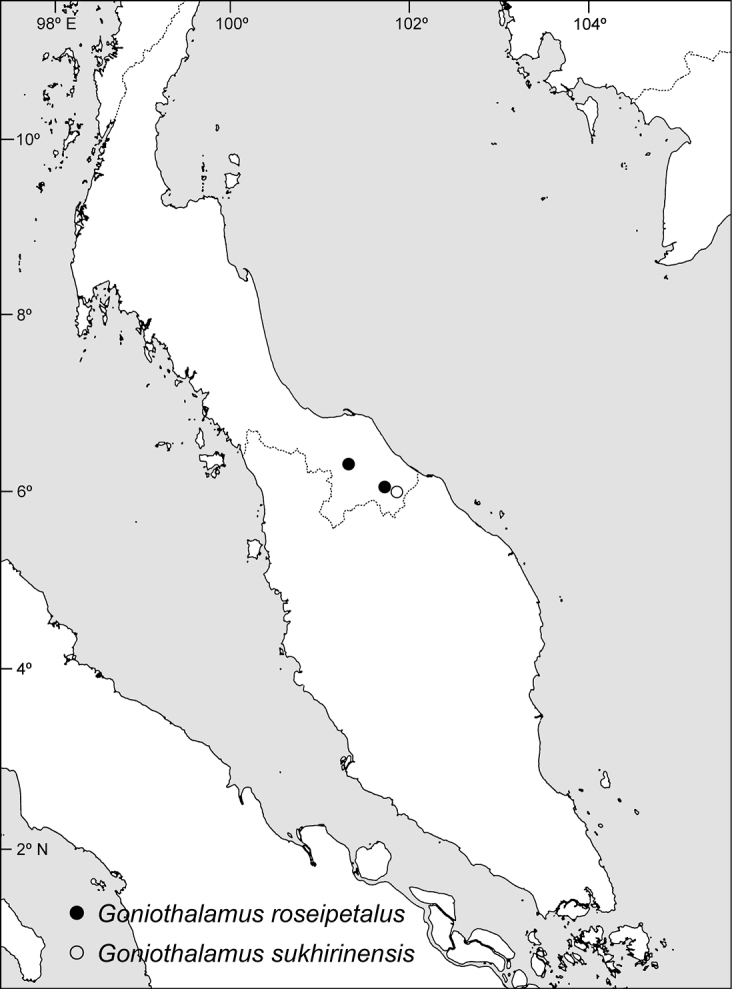
Distributions of *Goniothalamusroseipetalus* and *G.sukhirinensis*.

Endemic to Peninsular Thailand, where it occurs in Narathiwat and Yala Provinces (Fig. 4). Growing in shady and moist areas of tropical rainforests and forest margins between para-rubber plantations and remnant rainforests; 100–400 m alt.

#### Etymology.

In reference to the red pigmentation of the petals.

#### Local name.

Panan klip muang (ปาหนันกลีบม่วง) (general).

#### Additional specimens examined (paratypes).

**Thailand**: Yala Province, Bannang Sata, 350 m alt., 1 August 2020, *C. Leeratiwong 20*–*1684* (PSU); Narathiwat Province: Cha Nae District, Du Son Yo subdistrict, 100 m alt., 6 March 2021, *C. Leeratiwong 21*–*1705* (PSU).

#### Discussion.

Although *G.roseipetalus* is yet to be included in a molecular phylogenetic analysis, it shares several morphological similarities with species in a clade (nested within clade ‘A1a’ *sensu*Tang et al. 2015a, b) that comprises *G.loerzingii* R.M.K.Saunders, *G.macrophyllus*, *G.scortechinii*, *G.uvarioides* and *G.wrayi* King. These species were previously classified by Bân (1974) within Goniothalamussubgen.Goniothalamussect.Goniothalamus, and are characterised by their essentially glabrous vegetative shoots and petioles, percurrent tertiary leaf venation, generally fused sepals with distinct venation, short inner petals, apiculate staminal connectives, relatively few carpels per flower, thick-cylindrical pseudostyles with a broad, hairy stigma, and seeds with a hairy testa. Although *G.roseipetalus* shares most of these diagnostic characters, its stigmas are glabrous.

*Goniothalamusroseipetalus* is morphologically most similar to *G.scortechinii* and *G.uvarioides*. It differs from these species, however, as it generally has fewer secondary veins in its leaves (15–22 pairs, vs [18–]21–26[–32] in *G.scortechinii* and 24–35 in *G.uvarioides*), larger sepals (20–30 by 24–35 mm, vs 8–24 by 8–23 mm in *G.scortechinii* and 12–16 by 5–13 mm in *G.uvarioides*), and wider inner petals (8–11 mm, vs 5–8 mm in *G.scortechinii* and 7–8.5 mm in *G.uvarioides*). It also has wider outer petals (14–25 mm) than *G.scortechinii* (8–14 mm), and can be distinguished from *G.uvarioides* by reference to its smaller monocarps (8–15 by 7–9 mm, vs 31–44 by 15–18 mm) with a single seed (vs four or five seeds per monocarp) and shorter stipes (3–5 mm, vs 12.5–17.5 mm). *Goniothalamusroseipetalus* also resembles the widespread species *G.macrophyllus*, although the latter species has creamy-white petals.

*Goniothalamusroseipetalus* also resembles *G.calycinus* J. Sinclair, a species that is endemic to Terengganu in Peninsular Malaysia (Saunders 2003). *Goniothalamusroseipetalus* differs, however, in its flower position (with flowers borne on young or older branches in *G.calycinus*), larger sepals (only 4.5–17 by 4–17 mm in *G.calycinus*), wider outer petals (only 7–14 mm wide in *G.calycinus*), larger inner petals (only 8–14 by 5–7.5 mm in *G.calycinus*), and by the absence of a persistent calyx in fruits of *G.calycinus*.

### Goniothalamus sukhirinensis

Taxon classificationPlantaeMagnolialesAnnonaceae

Leerat., Chalermglin & R.M.K.Saunders sp. nov.

5FBB87FB-3873-581A-A547-6576F6E21FB0

urn:lsid:ipni.org:names:77221294-1

Figs 5 , 6

#### Diagnosis.

*Goniothalamussukhirinensis* resembles *G.macrophyllus* and *G.scortechinii*, but is distinguished by its densely hairy shoots, numerous secondary veins (32–40 pairs per leaf), generally longer pedicels (flowering: 12–18 mm; fruiting: 20–25 mm), larger outer petals (34–37 by 18–22 mm), larger monocarps (20–27 by 9–13 mm) that are densely hairy, and longer seeds (13–17 mm).

**Figure 5.**
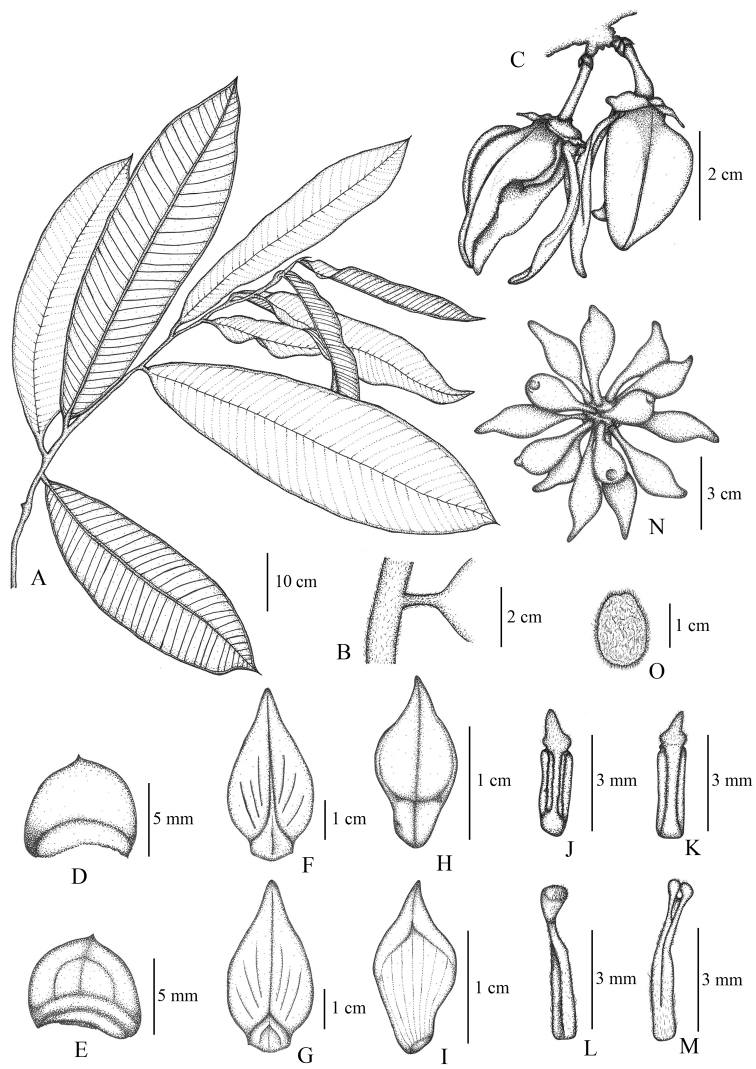
*Goniothalamussukhirinensis* sp. nov. **A** vegetative branch **B** petiole, showing base of leaf lamina **C** flowers **D** sepal (abaxial) **E** sepal (adaxial) **F** outer petal (abaxial) **G** outer petals (adaxial) **H** inner petal (abaxial) **I** inner petal (adaxial) **J** stamen (abaxial) **K** stamen (adaxial) **L** carpel (abaxial) **M** carpel (adaxial) **N** fruit, composed of separate monocarps **O** seed with hairy surface. Drawn by A. Somphrom from *C. Leeratiwong 21*–*1708* (PSU).

#### Types.

**Thailand**: Narathiwat: Sukhirin, Ban Yade village, Ma Mong subdistrict, 167 m alt., 6 March 2021, *C. Leeratiwong 21*–*1708* (holotype PSU; isotypes BKF, KKU).

**Figure 6.**
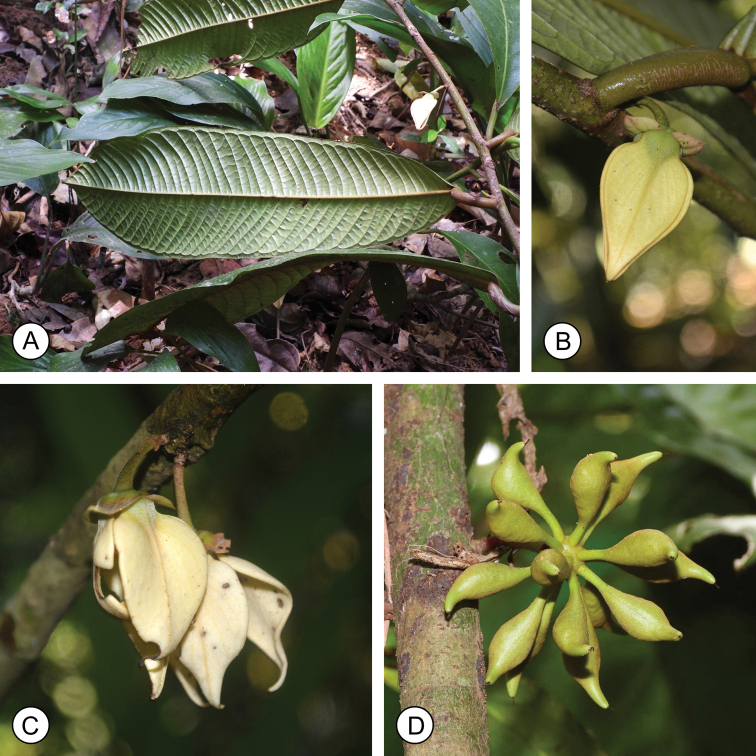
*Goniothalamussukhirinensis* sp. nov. **A** leaf (abaxial) **B, C** flowers **D** fruit. Photos by P. Chalermglin.

#### Description.

Shrubs to small trees, to 4 m. Young branches densely appressed-pubescent. Leaf laminas 28–50 by 7–16 cm, length/width ratio 3.1–4, (lanceolate-)oblong, apex generally acuminate to caudate (rarely acute to obtuse), acumen 7–20 mm long, base broadly cuneate, subcoriaceous, glabrous abaxially (sparsely hairy over midrib), sparsely pubescent adaxially (densely hairy over veins); midrib strongly prominent abaxially, sunken adaxially; secondary veins 32–40 pairs, plane adaxially; tertiary veins percurrent, distinct, lacking a ‘granular’ appearance abaxially; petioles 20–30 mm by 4–6 mm, densely pubescent. Flowers solitary or paired, often on main trunk (cauliflorous), rarely on older branches (ramiflorous), pendent; flowering pedicels 12–18 mm long, densely hairy; pedicel bracts ovate-triangular, 2.5–3 by 1–1.5 mm. Sepals greenish-pink, broadly ovate, 7–9.5 by 7.5–10 mm, basally connate (2.5–3 mm from base), apex acute, moderately hairy abaxially, sparsely hairy adaxially, venation indistinct. Outer petals greenish-yellow when young, whitish-yellow (green at claw) when mature, 34–37 by 18–22 mm with 3–5 mm-long claw, length/width ratio 1.6–1.9, fleshy, (lanceolate-)ovate, apex acuminate, densely hairy abaxially, moderately hairy adaxially with velutinous basal region facing apertures between inner petals, midrib raised adaxially, venation indistinct ab- and adaxially. Inner petals 13–15 by 7–8 mm with 2–3 mm long claw, length/width ratio 1.8–1.9, ovate-lanceolate, densely hairy abaxially, sparsely hairy distally adaxially, yellowish-green when young, pinkish-orange to reddish-brown when mature, apex acuminate, lacking a glabrous lasteral flange on the inner petal claws. Stamens numerous, oblong, 2.5–3.7 mm long; connectives apiculate, papillate. Carpels 11–20 per flower, ovary oblong, 2–3 mm long, with white hairs; stigma and pseudostyle 2–2.5 mm long, stigma funnel-shaped, hairy. Fruits sometimes with persistent calyx, immature fruits brownish-green, mature fruits not seen; fruiting pedicels 20–25 by 2–3.5 mm, sparsely hairy. Monocarps 5–14 per fruit, single-seeded, 20–27 by 9–13 mm, length/width ratio 2–2.7, (obovoid-)ellipsoid, apex apiculate, apicule 5–8 mm long, smooth, densely hairy, glossy, pericarp 1–2 mm thick, stipes 7–15 by 2–3 mm, densely hairy. Seeds 13–17 by 8–10 mm, length/width ratio 1.6–1.7, ellipsoid, testa densely villose, slightly rugose.

#### Phenology.

Flowering and fruiting in February and March (based on limited data).

#### Distribution and habitat.

Endemic to Narathiwat Province, Peninsular Thailand (Fig. 4). Growing in shady and moist areas of tropical rainforests; 167–200 m alt.

#### Etymology.

From the name Sukhirin, Narathiwat Province.

#### Local name.

Ratchakhru khao (ราชครูขาว) (Narathiwat).

#### Additional specimen examined (paratype).

**Thailand**: Narathiwat Province: Sukhirin District, Ban Yade village, Ma Mong subdistrict, 200 m alt., 28 February 2021, *C. Leeratiwong 21*–*1707* (PSU).

#### Discussion.

As with the previous species, *G.sukhirinensis* is yet to be included in a molecular phylogenetic analysis but has strong morphological affiliations with a clade that comprises *G.loerzingii* R.M.K.Saunders, *G.macrophyllus*, *G.scortechinii*, *G.uvarioides* and *G.wrayi* King (nested within clade ‘A1a’ *sensu*Tang et al. 2015a, b). The morphological characteristics of this clade are detailed under *G.roseipetalus*, above.

*Goniothalamussukhirinensis* resembles *G.macrophyllus* and *G.scortechinii*, but differs in several key characters: densely hairy shoots (vs glabrous to medium-hairy); numerous secondary veins (32–40 pairs per leaf, vs 12–23 in *G.macrophyllus* and [18–]21–26[–32] in *G.scortechinii*); generally longer flowering pedicels (12–18 mm, vs 5–11.5 mm in *G.macrophyllus* and 8–13 mm in *G.scortechinii*); larger outer petals (34–37 by 18–22 mm, vs 10–28 by 4.5–11.5 mm in *G.macrophyllus* and 20–33 by 8–14 mm in *G.scortechinii*); longer fruiting pedicels (20–25 mm, vs 7–19 in *G.macrophyllus* and 8–20 mm in *G.scortechinii*); larger monocarps (20–27 by 9–13 mm, vs 8–15 by 7.5–10 mm in *G.macrophyllus* and 9–18 by 6–10 mm in *G.scortechinii*) that are densely hairy (vs subglabrous to medium-hairy); and longer seeds (13–17 mm, vs 8.5–12 mm in *G.macrophyllus* and 8–11 mm in *G.scortechinii*). *Goniothalamussukhirinensis* also differs from *G.macrophyllus* as its leaves lack the fine ‘granular’ appearance of the latter species (due to the immersion of tertiary and higher-order veins: Saunders, 2002), and has longer monocarp stipes (7–15 mm, vs up to 1.8 mm in *G.macrophyllus*).

### Key to *Goniothalamus* species in Thailand (flowering specimens)

**Table:** 

1a	Stamen connective apex apiculate	**2**
2a	Young branches densely hairy to velutinous	**3**
3a	Leaf laminas 28–50 cm long, with 32–40 pairs of secondary veins; sepals 7–9.5 by 7.5–10 mm; outer petals 34–37 by 18–22 mm; inner petals 13–15 by 7–8 mm	***G.sukhirinensis* sp. nov.**
3b	Leaf laminas 50–76 cm long, with 24–32 pairs of secondary veins; sepals 30–40 by 28–30 mm; outer petals 60–80 by 30–40 mm; inner petals ca. 35 by ca. 17 mm	***G.cheliensis* H.H.Hu**
2b	Young branches glabrous to hairy	**4**
4a	Flowers in large fascicles, exclusively from woody tubercles at base of trunk	***G.ridleyi* King**
4b	Flowers solitary or in pairs, not exclusively from base of trunk	**5**
5a	Adaxial surface of outer petals with glabrous or sparsely hairy region facing apertures between inner petals	**6**
6a	Leaves with 24–35 pairs of secondary veins	***G.uvarioides* King**
6b	Leaves with 11–22 pairs of secondary veins	***G.tapis* Miq.**
5b	Adaxial surface of outer petals with velutinous region facing apertures between inner petals	**7**
7a	Flowering pedicels 20–37 mm long; carpels 50–100 per flower	***G.tortilipetalus* M.R.Hend.**
7b	Flowering pedicels 5–19 mm long; carpels 8–50 per flower	**8**
8a	Leaves with 9–12 pairs of secondary veins; stamen connective apex distinctly tapered; carpels 8–10 per flower	***G.tavoyensis* Chatterjee**
8b	Leaves with 12–26(–32) pairs of secondary veins; stamen connective apex not distinctly tapered; carpels 11–50 per flower	**9**
9a	Tertiary venation reticulate	**10**
10a	Sepals 11–18.5 mm long, 8–15.5 mm wide; outer petals 21–46 mm long, 4.5–18 mm wide, yellow; inner petal length/width ratio 3–6.5; stamens 95–120 per flower	***G.calvicarpus* Craib**
10b	Sepals 14–29 mm long, 12–26 mm wide; outer petals 36–104 mm long, 14–24 mm wide, green; inner petals length/width ratio 1.8–3.6; stamens 100–200 per flower	***G.griffithii* Hook.f. & Thomson**
9b	Tertiary venation percurrent	**11**
11a	Sepals 24–35 mm wide; outer petals 14–25 mm wide	***G.roseipetalus* sp. nov.**
11b	Sepals 4–23 mm wide; outer petals 4.5–14 mm wide	**12**
12a	Leaf laminas (sub-)coriaceous, with fine “granular” texture abaxially (due to immersion of tertiary and lower order veins); leaves with 12–23 pairs of secondary veins	***G.macrophyllus* (Blume) Hook.f. & Thomson**
12b	Leaf laminas papyraceous, without fine ‘granular’ texture abaxially; leaves with (18–)21–26(–32) pairs of secondary veins	***G.scortechinii* King**
1b	Stamen connective apex truncate	**13**
13a	Inner petal claws with distinct glabrous lateral flange	**14**
14a	Flowering pedicels 7–23 mm long; stigma subulate	**15**
15a	Outer petals 23–43 mm long, 12–23 mm wide; carpels 40–100 per flower	***G.sawtehii* C.E.C.Fisch.**
15b	Outer petals 10.5–32 mm long, 5.5–17.5 mm wide; carpels 10–54 per flower	***G.undulatus* Ridl.**
14b	Flowering pedicels 2–11.5 mm long; stigma fusiform or funnel-shaped	**16**
16a	Young branches densely hairy to velutinous	***G.tamirensis* Pierre ex Finet & Gagnep.**
16b	Young branches glabrous to hairy	**17**
17a	Flowering pedicels 5–11.5 mm long; sepal venation generally indistinct; outer petals 12.5–73 mm long; stigma fusiform	***G.laoticus* (Finet & Gagnep.) Bân**
17b	Flowering pedicels 2–6 mm long; sepal venation distinct; outer petals 8.5–39 mm long; stigma funnel-shaped	**18**
18a	Leaf laminas 8–14 cm long, 1.5–4 cm wide; petioles 3.5–7 mm long; sepals 3–9 mm long, 3.5–6 mm wide; outer petals 8.5–15 mm long, 3.5–8 mm wide, very densely hairy ab- and adaxially; inner petals 6.5–10 mm long, 3–4.5 mm wide; ovary glabrous	***G.elegans* Ast**
18b	Leaf laminas 12.5–24.5 cm long, 4–8.5 cm wide; petioles 5–15 mm long; sepals 7.5–12.5 mm long, 5.5–11 mm wide; outer petals 23–39 mm long, 7–15 mm wide, glabrous to hairy ab- and adaxially; inner petals 10–16 mm long, 5.5–9 mm wide; ovary sparsely hairy	***G.latestigma* C.E.C.Fisch.**
13b	Inner petal claws without glabrous lateral flange	**19**
19a	Tertiary leaf venation generally reticulate; outer petals with velutinous region at base of adaxial surface (facing aperture between inner petals); inner petals velutinous adaxially	**20**
20a	Sepals 2.5–3.5 mm long, basally connate, venation indistinct; outer petal venation distinct; stigma fusiform	***G.repevensis* Pierre ex Finet & Gagnep.**
20b	Sepals 4.5–19 mm long, free, venation distinct; outer petal venation indistinct; stigma subulate or funnel-shaped	**21**
21a	Young branches glabrous; outer petal length/width ratio 3.4–5.2	***G.expansus* Craib**
21b	Young branches very sparsely to densely hairy; outer petal length/width ratio 1.6–3.8	**22**
22a	Leaves with fine “granular” texture abaxially (due to immersion of tertiary and lower order veins); flowers slightly supra-axillary	***G.rotundisepalus* M.R.Hend.**
22b	Leaves without fine “granular” texture abaxially; flowers axillary	***G.tenuifolius* King**
19b	Tertiary leaf venation percurrent; outer petals with glabrous or sparsely hairy region at base of adaxial surface (facing aperture between inner petals); inner petals glabrous to densely hairy adaxially	**23**
23a	Flowering pedicels 20–48 mm long; sepals 7–15 mm long; outer petals 68–113 mm long, 28–63 mm wide	***G.giganteus* (Wall. ex) Hook.f. & Thomson**
23b	Flowering pedicels 8–16(–21) mm long; sepals 2–8 mm long; outer petals 16–50(–62) mm long, 7–22(–32) mm wide	**24**
24a	Inner petals glabrous adaxially; ovaries densely hairy	***G.malayanus* Hook.f. & Thomson**
24b	Inner petals (densely) hairy adaxially (sometimes glabrous towards base); ovaries glabrous to sparsely hairy	**25**
25a	Leaf laminas 23–32 cm long, with 14–21 pairs of secondary veins; stamens 50–160 per flower; carpels 4–11 per flower	***G.aurantiacus* R.M.K.Saunders & Chalermglin**
25b	Leaf laminas 17–25.5 cm long, with 13–16 pairs of secondary veins; stamens ca. 180–200 per flower; carpels ca. 18–20 per flower	**26**
26a	Flowering pedicels densely hairy; outer petals densely hairy abaxially, very densely hairy adaxially, venation indistinct; inner petals very densely hairy abaxially	***G.maewongensis* R.M.K.Saunders & Chalermglin**
26b	Flowering pedicels very sparsely hairy; outer petals subglabrous abaxially, glabrous adaxially, venation distinct; inner petals sparsely hairy abaxially	***G.rongklanus* R.M.K.Saunders & Chalermglin**

### Key to *Goniothalamus* species in Thailand (fruiting specimens)

**Table:** 

1a	Adaxial surface of leaves with very prominent secondary veins	**2**
2a	Leaf laminas 50–76 cm long, 13–22 cm wide, with 24–32 pairs of secondary veins; leaf midrib densely hairy to velutinous; petioles 17–30 mm long, velutinous; monocarps densely hairy	***G.cheliensis* H.H.Hu**
2b	Leaf laminas 12.5–39.5 cm long, 3.5–9.5(–11.5) cm wide, with 10–22 pairs of secondary veins; leaf midrib glabrous to sparsely hairy; petioles 4–16 mm long, glabrous to hairy; monocarps glabrous to hairy	**3**
3a	Monocarps distinctly warty	***G.giganteus* (Wall. ex) Hook.f. & Thomson**
3b	Monocarps smooth or finely rugulose	**4**
4a	Fruits restricted to trunk; fruiting pedicels 19–36 mm long	***G.tortilipetalus* M.R.Hend.**
4b	Fruits not restricted to trunk; fruiting pedicels 10–19 mm long	**5**
5a	Tertiary leaf venation percurrent; fruits without persistent calyx; monocarps 16–40 mm long, 8–13(–17) mm wide; seeds 13–20 mm long, with (sparsely) hairy testa	***G.malayanus* Hook.f. & Thomson**
5b	Tertiary leaf venation reticulate; fruits with persistent calyx; monocarps 10–14 mm long, 7–8 mm wide; seeds 10–12 mm long, with glabrous testa	**6**
6a	Monocarps red; seeds slightly rugose	***G.calvicarpus* Craib**
6b	Monocarps yellow-brown; seeds smooth	***G.griffithii* Hook.f. & Thomson**
1b	Adaxial surface of leaves with impressed or only slightly prominent secondary veins	**7**
7a	Tertiary leaf venation percurrent	**8**
8a	Monocarps apiculate	**9**
9a	Young branches densely hairy; leaves with 32–40 pairs of secondary veins; fruiting pedicels 20–25 mm long; monocarps 20–27 mm long, densely hairy; monocarp stipes 7–15 mm long	***G.sukhirinensis* sp. nov.**
9b	Young branches glabrous to hairy; leaves with 12–23 pairs of secondary veins; fruiting pedicels 7–20 mm long; monocarps 8–15 mm long (very) sparsely hairy; monocarp stipes up to 5 mm long	**10**
10a	Fruits without persistent calyx	***G.macrophyllus* (Blume) Hook.f. & Thomson**
10b	Fruits with persistent calyx	***G.roseipetalus* sp. nov.**
8b	Monocarps not apiculate	**11**
11a	Monocarps 7–18 mm long, 6–10 mm wide	**12**
12a	Leaf laminas 22–40(–50) cm long, 5.5–12(–19.5) cm wide, with (18–)21–26(–32) pairs of secondary veins	***G.scortechinii* King**
12b	Leaf laminas 8–20 cm long, 2.5–6 cm wide, with 8–14 pairs of secondary veins	***G.tenuifolius* King**
11b	Monocarps 16–56 mm long, 13–29 mm wide	**13**
13a	Leaf laminas 32–48 cm long, 7–15.5 cm wide, with 24–35 pairs of secondary veins; fruits without persistent calyx	***G.uvarioides* King**
13b	Leaf laminas 14–32 cm long, 3.5–9.5 cm wide, with 11–21 pairs of secondary veins; fruits with persistent calyx	**14**
14a	Fruits restricted to base of trunk; fruiting pedicels 30–130 mm long	***G.ridleyi* King**
14b	Fruits not restricted to base of trunk; fruiting pedicels 10–22 mm long	**15**
15a	Monocarps subsessile or with stipe ≤ 1 mm long; seeds 18–19 mm wide, rugose	***G.maewongensis* R.M.K.Saunders & Chalermglin**
15b	Monocarp stipes 2–8 mm long; seeds 13–17 mm wide; smooth to slightly rugulose	**16**
16a	Leaf laminas 23–32 cm long, with 14–21 pairs of secondary veins; fruiting pedicels ca. 14 mm long; seeds 19–27 mm long, length/width ratio 1.5–1.7	***G.aurantiacus* R.M.K.Saunders & Chalermglin**
16b	Leaf laminas 17–25 cm long, with 13–16 pairs of secondary veins; fruiting pedicels 16–22 mm long; seeds 15–22 mm long, length/width ratio 1.1–1.5	***G.rongklanus* R.M.K.Saunders & Chalermglin**
7b	Tertiary leaf venation reticulate	**17**
17a	Monocarps 22–56 mm long, 14–21 mm wide, with prominent longitudinal ridge; up to 7 seeds per monocarp	***G.laoticus* (Finet & Gagnep.) Bân**
17b	Monocarps 8.5–26 mm long, 6–11 mm wide, without longitudinal ridge; 1 or 2 seeds per monocarp	**18**
18a	Fruiting pedicels 8–20 mm long	**19**
19a	Young branches (very densely) hairy	**20**
20a	Leaf laminas glossy adaxially; monocarp stipes 6.5–16.5 mm long; seeds 9–11.5 mm long	***G.undulatus* Ridl.**
20b	Leaf laminas matt adaxially; monocarp stipes 3–6 mm long; seeds 11–14 mm long	***G.sawtehii* C.E.C.Fisch.**
19b	Young branches glabrous to hairy	**21**
21a	Leaf lamina tertiary venation (clearly) distinct	***G.expansus* Craib**
21b	Leaf lamina tertiary venation indistinct to ± distinct	**22**
22a	Leaf laminas with 9–12 pairs of secondary veins; monocarps greenish-yellow, ca. 19 mm long; seeds 17–18 mm long	***G.tavoyensis* Chatterjee**
22b	Leaf laminas with 11–16 pairs of secondary veins; monocarps (dark) red, 10–14 mm long; seeds 9–12 mm long	***G.tapis* Miq.**
18b	Fruiting pedicels 4–8 mm long	**23**
23a	Monocarps greenish-yellow	**24**
24a	Young branches hairy; leaf laminas 12.5–24.5 cm long, 4–8.5 cm wide; monocarps smooth; seeds ca. 17.5 mm long	***G.latestigma* C.E.C.Fisch.**
24b	Young branches (very) sparsely hairy; leaf laminas 10.5–13.5(–16) cm long, 3–5 cm wide; monocarps very finely rugulose; seeds ca. 9.5 mm long	***G.repevensis* Pierre ex Finet & Gagnep.**
23b	Monocarps red	**25**
25a	Fruits without persistent calyx; seeds subglabrous to hairy	***G.tapis* Miq.**
25b	Fruits with persistent calyx; seeds glabrous	**26**
26a	Leaf laminas 1.5–4 cm wide, ± glossy adaxially	***G.elegans* Ast**
26b	Leaf laminas 3.5–8.5 cm wide, (±) matt adaxially	**27**
27a	Monocarps glabrous; monocarp stipes 4.5–13 mm long	***G.tamirensis* Pierre ex Finet & Gagnep.**
27b	Monocarps very sparsely hairy; monocarp stipes 2–3 mm long	***G.rotundisepalus* M.R.Hend.**

## Supplementary Material

XML Treatment for Goniothalamus roseipetalus

XML Treatment for Goniothalamus sukhirinensis
